# Optimizing preparedness and response for circulating vaccine derived poliovirus (cVDPV) outbreaks in the Eastern Mediterranean Region with a particular focus on cVDPV2

**DOI:** 10.3389/fpubh.2025.1663402

**Published:** 2025-10-10

**Authors:** Nadine Haddad, Najwa Jarour, Hemant Shukla, Asnakew Tsega, Irfan Akbar, Ghada Mahmoud Qasim Shahrour, Subhranshu Sekhar Kar, Mohannad Al Nsour, Magid Gunaid, Yousef Khader

**Affiliations:** ^1^The Eastern Mediterranean Public Health Network (EMPHNET), Amman, Jordan; ^2^World Health Organisation Regional Office for the Eastern Mediterranean, Cairo, Egypt; ^3^United Nations Agency for Children, Middle East and North African Regional Office (MENARO), Amman, Jordan; ^4^RAK Medical and Health Sciences University, Ras Al Khaimah, United Arab Emirates

**Keywords:** CVDPV (circulating vaccine derived poliovirus), polio, preparedness and response, Eastern Mediterranean (EM), acute flaccid paralyses

## Abstract

Efforts to achieve polio eradication in the Eastern Mediterranean Region (EMR) are confronted by several barriers stemming from the volatile security and political instability in several countries, to social contextual challenges such as inaccessible and mobile/displaced population, to logistic issues hindering optimal implementation of immunization and surveillance interventions. To further understand these challenges and emphasize improvement areas, the Eighth Regional Conference of the Eastern Mediterranean Public Health Network (EMPHNET), that was held in Amman, Jordan from September 15–18, 2023, featured a workshop focused on scaling up outbreak preparedness and response efforts. A variety of relevant experts and audiences from outbreak and at-risk countries in the EMR described their capacities, best practices and lessons learned from their national experiences. Overall recommendations were outlined, aiming to enhance preparedness and response to cVDPV outbreak in target countries and in the region overall.

## Introduction

Despite significant progress in polio eradication over the past decade, several challenges still prevent its complete achievement, particularly in the Eastern Mediterranean Region (EMR) (1). EMR remains the only World Health Organization (WHO) region still endemic for wild poliovirus and experiences outbreaks of circulating vaccine derived poliovirus (cVDPV) (2), lagging behind the other five regions declared polio-free between 1994 and 2020: Pan-America (3), Western Pacific (4), European (5), South-East Asia (6), and Africa (7).

Pol3 immunization coverage in EMR reached 80% in 2022, though disparities between countries persist (8). With the persistence of zero dose and under-immunized children, EMR countries are hence at risk of Polio and cVDPV outbreaks (9). Afghanistan and Pakistan, remain endemic as Wild polio type 1 (WP1) cases continue to be reported (10). As of September 1st, 2024, Afghanistan reported 18 WP1 cases, up from 5 cases during the same period in 2023. A similar rise was observed in Pakistan, where 16 WP1 cases were reported in 2024, compared to 2 cases in 2023 (11).

Since OPV2 was withdrawn in 2016, global and regional cVDPV2 circulation has increased. Yemen reported cVDPV2 alongside a cVDPV1 outbreak that paralyzed 35 children between 2019 and 2021 (12). In Egypt, environmental samples from Giza collected since 2022 in Egypt were positive for vaccine-derived poliovirus type 2 (VDPV2) (13). Afghanistan and Pakistan face dual WP1 and cVDPV2 transmission (14, 15), while Somalia experienced cVDPV2 and cVDPV3 outbreaks, the latter ending in 2021 (16). Sudan had repeated cVDPV2 outbreaks between 2022 and 2024 (17, 18).

The global rise in cVDPV2 cases was driven by factors common to several countries: declining mucosal immunity to type 2 poliovirus among young children born after the switch to inactivated polio vaccine (IPV) on one hand, and limited global vaccine stockpile availability that restricts the scope of supplementary immunization activities (SIAs) on the other hand (19, 20). Particularly for EMR countries, cVDPV2 outbreaks and transmission are further exacerbated by low coverage of essential immunization with IPV, regional migration patterns facilitating the virus’s movement between populations, delays in detection and implementation of outbreak responses, and inconsistent quality in SIAs during outbreak responses (1).

To achieve a polio-free world, the Global Polio Eradication Initiative (GPEI) Polio Eradication Strategy 2022 prioritizes two overarching goals: to permanently interrupt all poliovirus transmission in the final WPV-endemic countries of Afghanistan and Pakistan; and, to stop cVDPV transmission and prevent outbreaks in non-endemic countries (1).

In response, the Eighth Regional Conference of the Eastern Mediterranean Public Health Network (EMPHNET), held in Amman, Jordan (Sept 15–18, 2023), hosted a workshop on enhancing outbreak preparedness and response. It reviewed the epidemiology of cVDPV—especially cVDPV2 in the EMR—and addressed pandemic-related setbacks, surveillance gaps, and health system challenges, identifying priority actions to strengthen regional cVDPV outbreak preparedness and response.

## Workshop description

### Target audience

The workshop targeted managers and teams of Expanded Program of Immunization (EPI)/PEI managers and teams, Acute Flaccid Paralysis (AFP) and/or integrated surveillance officers, Rapid Response Teams managers, Field Epidemiology Training Program (FETP) residents and graduates, and GPEI partners. Forty participants from outbreak countries (Egypt, Yemen, Somalia and Sudan) or countries at risk (Iraq, Libya and Lebanon) attended.

### Workshop content

The workshop started with a review of Strategic Advisory Group of Experts (SAGE) for polio vaccination for children under five, including routine immunization, outbreak response, and preventive campaigns this was followed by an overview of country-specific immunization coverage and polio transmission risk in the EMR and presentation of the two goals of the GPEI Strategy. Epidemiological updates on Polio/cVDPV outbreaks were shared, highlighting challenges faced in responding to cVDPV outbreaks in EMR.

Participants from outbreak countries presented their national data and described their response to the current or previous cVDPV2 outbreaks, with a particular focus on local challenges and gaps. Next, participants from at-risk countries presented their preparedness and response plans focusing on their capacity to detect importation and response to cVDPV2 transmission. Lastly, priority recommendations and action points were provided. The workshop was moderated by experts from WHO-EMRO, United Nations International Children’s Emergency Fund (UNICEF) and the Centers for Disease Control and Prevention in Atlanta (CDC).

## Findings

### Inadequate immunization coverage in the Eastern Mediterranean Region

Notable discrepancies in Diphtheria tetanus toxoid and pertussis (DTP3)/Oral Polio Virus 3 coverage exist within the EMR countries. Countries with high coverage level (>90%) include Saudi Arabia, Kuwait, UAE, Oman, Iran, Qatar, Tunisia, Morocco, Iraq and Jordan (despite a fluctuating coverage) and Egypt (with a low subnational level coverage). Countries with suboptimal coverage (70–90%) include Lebanon, Libya, Syria, Palestine, Pakistan, whereas countries with low coverage (<70%) include Somalia, Afghanistan, Sudan, Yemen and Djibouti (21). Inadequate coverage rates in some countries put the whole EMR at risk due to the ongoing circulation of polio in the region and globally. Countries with low coverage countries have ongoing polio transmission already, and those with sub-optimal coverage are at risk of sustained polio transmission in case of polio importation. Moreover, AFP surveillance indicators varied between EMR countries, highlighting different capacities in detection and adequate investigation of cases (22).

In line with SAGE recommendations and EMR immunization profiles, participants emphasized the need for countries to thoroughly assess immunization and epidemiological data at all levels. This helps identify low-coverage areas, silent zones, and high-risk pockets before outbreaks occur. Strengthening routine immunization is especially critical in countries with sub-optimal or declining coverage due to the pandemic or ongoing conflicts (Yemen, Somalia, Afghanistan, Sudan, Syria, Libya, Palestine, Djibouti, Lebanon) and specifically explore and address the sub-national immunization coverage in Egypt. Revising the immunization schedule was advised for Palestine and Sudan with respect to IPV, and for Lebanon with respect to an earlier administration of the bivalent Oral Polio Vaccine (bOPV).

### Pillars of preparedness and response to polio outbreaks

Participants emphasized the need for strengthening the outbreak response preparedness through the national Polio outbreak response preparedness plans and continuously revising it through a series of simulation exercises. Additionally, the participants welcomed the support of the Incident Management Support Team (IMST) and regional member states in mounting response by providing technical support, field surge support, finance/logistics and periodic outbreak response assessment.

In terms of response, there was a consensus between the audience on continuation of review of the outbreak response plan following Outbreak Response Assessment (OBRA). This includes conducting periodic external assessments for 120 days and 6 months following the confirmation of the outbreak. Quarterly assessments are also conducted till 6 months after the last case was detected. Afterwards, a final external assessment is conducted focusing on surveillance and other activities to maintain the polio-free status of the country. On the other hand, the importance of conducting a detailed review of the communications plans for subsequent phases, and tracking progress made and/or support needed was also emphasized to close any remaining gaps.

### Experience from outbreak countries: Yemen, Sudan and Somalia

The experiences of Yemen, Sudan and Somalia in responding to the cVPDV outbreaks were showcases as examples to extract their response pillars, challenges and best practices.

In Yemen, 274 cVDPV2 cases were reported between August 2021 and June 2024 in both Northern and Southern governorates, as per their national published and unpublished figures. The implemented national response plan involved timely and close coordination between local, regional and international stakeholders and relied on conducting risk assessment, enhancing human and environmental surveillance (ES), strengthening vaccination and conducting SIA as well as strengthening risk communication. In terms of SIAs, three consecutive immunization campaigns were conducted in in the southern region of Yemen between January and June 2022, followed by one campaign in 2023 using the Trivalent oral polio vaccine (tOPV), and two rounds were conducted in 2024 using the novel OPV type 2 (nOPV2), which effectively halted the circulation of cVDPV2 (23). The vaccination strategy was continuously revised to respond to changing epidemiological contexts, with a strong political backing from high-level leadership and key figures On the other hand, the active engagement of local communities in social mobilization activities, awareness and vaccination efforts compensated the shortage of health facilities providing vaccination and contributed to a significant reduction in vaccine hesitancy and refusal cases.

Nevertheless, political division between Northern and Southern governments and the two ministries of Public Health and populations resulted a lack of coordination at all levels. In the Northern governorates, the protracted complex emergency and volatile security resulted in the continuity of cVDPV2 and Measles outbreaks. Planned immunization activities were not implemented in this part of the country, which was further exacerbated by a high refusal rate which led to the cessation of routine vaccine distribution.

In Somalia, approximately 1.3 million children in 2023 are either under/unimmunized children and hence missed by the immunization program. Outbreaks of measles, diphtheria, whooping cough, and neonatal tetanus were also reported (24). Simultaneously, the country has been facing cVDPV2 outbreak since 2017 while the last WPV case was reported in 2014 (25).

As part of its response for polio eradication, the Somalia Emergency Action Plan (SEAP)-3.

supports polio eradication through high-level advocacy, improving health service coverage and immunization quality, enhancing cross-border and national coordination, and leveraging humanitarian efforts to reach missed children. The plan also focuses on optimizing cold chain and vaccine management, strengthening surveillance, and boosting community engagement and awareness for campaigns and routine immunization. Somalia introduced nOPV2 vaccine in May 2023 and in 2024, FMoH issued policy guidance on the expansion of the BIG Catch-Up (BCU) campaign to target age-group up to 5 years, and which integrated polio eradication (26).

Nevertheless, these national efforts still confront numerous challenges given the country’s political, security and economic situation. Around 17% (472,000) of targeted children live in inaccessible areas. The access to routine immunization is also a major issue: A Harmonized Health Facility Assessment recently conducted indicates that only immunization services are available at 62% of public hospitals and 68% of health centres, but only a small proportion (17%) of primary health units. On the other hand, as only 47% of the population live within 10 kilometers of an immunization centre, more reliance is placed on outreach and SIAs, thus resulting in unsustainable high operational costs (27). Additionally, several logistic operational challenges hinder the timely and full implementation of BCU and response to multiple diseases outbreaks; these challenges include inadequate resources to procure traditional RI vaccines as well as sub-optimal cold chain capacity. On the other hand, the National Immunization Days (NIDs) for NID polio campaigns were postponed due to nOPV2 global supply issues. Limited lab capacity delays outbreak response, and staff shortages hinder managing VPD outbreaks; priorities include improving operations, community engagement, cross-border collaboration, and tech-enabled microplanning. In Sudan, six cVDPV2 isolates were detected in Port Sudan in January 2024, followed by two Sabin-like AFP cases in Gedaref in May. Within 24 h, the Ministry of Health activated national and state response committees, engaging key stakeholders and launching the preparedness plan. Within 72 h, enhanced surveillance, RI strengthening, mop-up campaigns, and health promotion began. By 14 days, staff training and clinician sensitization were completed, and environmental sampling was conducted in border states. Two SIA rounds followed from March to August 2024, along with intensified RI activities (28).

Strengthening cross-border collaboration was also a priority. A cross-border coordination meeting Sudan and Egypt was conducted in November 2024 during which a joint cross-border Coordination Management Body (CMB) was established, with assigned national and sub-national focal points, to follow up on the implementation of recommendations and monitoring/evaluation.

Sudan faced challenges similar to Somalia, including delays in lab testing due to security issues and specimen transport. Access to nomadic and displaced populations was difficult, compounded by poor connectivity. Notably, Sudanese women played a crucial role, overcoming floods, difficult terrain, and cultural barriers to deliver vaccinations.

Financial sustainability was a major issue, with donor phasing out leading to human resource losses. However, the Field Epidemiology Training Program (FETP) helped fill staffing gaps effectively. Rumor investigation and outbreak documentation require improvement.

Strengthening community-based reporting, involving locals in awareness sessions, and improving micro-planning at local level are essential to reach high-risk areas, track defaulters, and close immunity gaps.

### Experience from countries at-risk: Lebanon, Libya, Iraq

The preparedness of at-risk countries such as Lebanon, Libya and Iraq was described on the light of epidemiological situation, ES activities, best practices and challenges.

The last WPV case in Lebanon was detected in 2003 following importation and contained timely. Despite its polio-free status, the country remains at risk of importation and sustained circulation due to the refugees and internally displaced population as well as the immunity gaps accumulating over the past years (29).

Surveillance and cross-border collaboration have improved through awareness sessions and updated guidelines. Environmental sampling expanded to nine sites since 2021. Capacity building included two Public Health Empowerment Program for surveillance and Polio officers (PHEP-SPO) cohorts training about 50 surveillance officers. Challenges remain in stakeholder coordination, worsened by high staff turnover due to brain drain.

As per the Ministry of health, the prospective steps include printing and disseminating the revised AFP surveillance guideline and SOPs, followed by maintaining refresher training and sensitization sessions. The preparedness plan should be regularly revised and tested via simulation exercises with stakeholder involvement. Programmatic improvements are needed for timely reporting and integrated polio surveillance. Expanding PHEP-SPO cohorts can support staff capacity building, incentivization, and retention.

Libya achieved Polio elimination in 1991, yet the ongoing risk of importation and cVDPV outbreaks remain high due to the cross-border movement. The national preparedness plan includes the main core pillars, namely in terms of surveillance and monitoring, RCCE, logistic and supply chain management, vaccination campaign, capacity building as well as coordination and governance. This plan was tested and validated in a simulation exercise supported by WHO in 2023 (30). Libya enhanced AFP detection by improving routine and event-based surveillance, laboratory capacity, technical guidelines, and staff training. Efforts included developing IDSR for better data use, launching the RASED platform for timely case reporting, and ongoing training on cVDPV response. Key challenges remain, including staff turnover, delayed incentives, the shift to digital reporting, weak coordination, outdated cold chain inventory, and limited advocacy for emergency use authorization.

Iraq undertook major reforms to enhance polio eradication efforts. Despite fluctuating non-Polio AFP rate and stool sample adequacy over the past years, most of AFP surveillance indicators remained beyond the certification standards (31). Since the establishment of ES in 2022, the National Polio Lab strengthened its ES sampling network and maintained high standards through regular accreditations, proficiency tests, and staff training (32, 33).

The National Polio Preparedness and Response Plan (NPPOPR) is updated annually, with biannual risk assessments and ongoing district-level evaluations. Updated annually, with ad-hoc reviews, the plan comprises the following pillars: Country Background Information, Management & Accountability, Risk Assessment & Response Plan, VDPV2 Response Protocols, and Response Assessment.

Key initiatives were implemented following the AFP review conducted in 2022, including capacity building for provincial surveillance officers, supervisory visits focusing on gender mainstreaming, and EWARN workshops for outbreak detection in IDP and refugee camps (34). National guidelines were updated to global standards and tested via simulation exercises. International stakeholder engagement was strengthened through meetings and field investigations. Outreach to zero-dose children continued, while DHIS2 use and coordination with media and hospitals improved community engagement.

As of January 2024, Iraq has fully transitioned polio management to its government, ending external financial support (35). This shift aligns with the global strategy for a sustainable, government-led approach. Currently, the aim is to integrate polio surveillance with other vaccine-preventable diseases to streamline processes. Several key areas require improvement. First, communication between the Central Public Health Laboratory (CPHL) and the Ministry of Health is inefficient, with unclear coordination with GPEI partners. The overlapping roles and unclear outbreak management in the Incident Management System (IMS) require further clarification. Financially, the plan lacks defined funding sources for outbreak response in addition to unclear details about the human resources, training and monitoring. Communication and social mobilization efforts lack differentiation between awareness and vaccination. Epidemiological examination and National Polio Laboratory (NPL) improvements need further elaboration, and comprehensive immunization response details are necessary, especially for type 2 outbreaks.

## Discussion

The final phase of polio eradication in the EMR is most challenging due to political instability and weak health systems, with shared obstacles across countries including insecurity, inaccessible populations, cross-border mobility, logistical issues, and high staff turnover; sustained efforts at all levels remain crucial (36).

Priority recommendations for both outbreak and at-risk countries include strengthening RI programs with high IPV coverage, introducing IPV2 into the EMR schedule, and enhancing RI and surveillance in at-risk areas using community-focused approaches for mobile populations. High-quality campaigns are essential in outbreak countries, while prevention campaigns are needed in at-risk areas. Improving RI microplans is also critical for better campaign quality and accurate mobile population estimates. The choice of the vaccine in-use and the immunization practice needs to consider the epidemiological and global recommendations, projected modellings and other countries’ practice (37). In all settings, immunization outreach activities need to promote a holistic approach for the Prevention of Sexual Exploitation, Abuse & Harassment (PRSEAH) (38).

Strengthening AFP surveillance through sub-national analysis and RI coordination enables timely cVDPV detection and response, while expanding and regularly reviewing environmental surveillance ensures better risk assessment; amid ongoing challenges, retaining skilled staff through capacity building—especially via PHEP-SPO—is essential for effective preparedness.

Countries should strengthen EOC leadership to regularly update national polio preparedness plans using current data, ensuring coordinated, agile responses—especially following Polio Outbreak Simulation Exercises (POSE) exercises.

## Conclusion

Achieving polio and cVDPV interruption in EMR requires coordinated national, regional, and global efforts. Political and security instability remains a major barrier, limiting immunization coverage and leaving vulnerable populations unreached. While challenges are shared across outbreak and at-risk countries, community-centered approaches tailored to local contexts are vital to boost vaccine demand and trust.

Enhanced surveillance must complement immunization efforts, supported by strong coordination for timely detection and response. National preparedness and response plans should be regularly reviewed with regional and global partners to align with international recommendations. Africa’s success in eradicating polio highlights the value of sustained vaccination, robust surveillance, and strong political and community engagement in preventing resurgence.

## Data Availability

The original contributions presented in the study are included in the article/supplementary material, further inquiries can be directed to the corresponding author.
